# Diversity and Antimicrobial Activities of *Actinobacteria* Isolated from Mining Soils in Midelt Region, Morocco

**DOI:** 10.1155/2023/6106673

**Published:** 2023-01-24

**Authors:** Soumia Ait Assou, Jaouad Anissi, Khalid Sendide, Mohammed El Hassouni

**Affiliations:** ^1^Biotechnology, Environment, Agri-Food and Health Laboratory, Faculty of Sciences Dhar El Mahraz, Sidi Mohamed Ben Abdellah University, BP. 1796, Atlas, Fez, Morocco; ^2^School of Engineering BIOMEDTECH, Euro-Mediterranean University of Fez, Meknes, BP 51, Fez, Morocco; ^3^Laboratory of Biotechnology, School of Science and Engineering, Al Akhawayn University in Ifrane, P.O. Box 104, Ifrane, Morocco

## Abstract

Multidrug-resistant bacteria have emerged as a serious global health threat that requires, more than ever before, an urgent need for novel and more effective drugs. In this regard, the present study sheds light on the diversity and antimicrobial potential of *Actinobacteria* isolates in mining ecosystems. We have indeed investigated the production of bioactive molecules by the *Actinobacteria* isolated from abandoned mining areas in Midelt, Morocco, where average contents of lead (Pb) and cadmium (Cd) are higher than normal world levels. One hundred and forty-five* Actinobacteria* isolates were isolated and characterized based on morphological, chemotaxonomical, biochemical, and molecular data. Most of the 145 isolates were identified as *Streptomyces*. Isolates affiliated to the genera *Amycolatopsis*, *Lentzea*, *Actinopolymorpha*, and *Pseudonocardia* were also found. Antimicrobial producing potentials of *Actinobacteria* isolates were assessed against eight test microorganisms Gram^+^ and Gram^−^ bacteria and yeast. Out of 145 isolates, 51 showed antimicrobial activities against at least one test microorganism. 31 isolates inhibited only bacteria, 7 showed activity against bacteria and *Candida albicans*, and 13 displayed activity against *C*. *albicans* solely. Our findings suggest that *Actinobacteria* isolated from natural heavy metal ecosystems may be a valuable source of novel secondary metabolites and therefore of new biotechnologically promising antimicrobial compounds.

## 1. Introduction

*Actinobacteria* are a group of Gram-positive filamentous bacteria with high G+C DNA content and are ubiquitously found in aquatic and terrestrial environments [1]. These bacteria represent the most renowned group of microorganisms for the production of bioactive compounds with diversified chemical structures [2, 3]. They are well known for antibiotics and antitumor agents production and are considered the source of about 45% of the obtained molecules from the microbial origin [4–6]. Among *Actinobacteria*, *Streptomyces* is still considered the best important source of bioactive secondary metabolites [6].

Since the discovery of streptomycin from *Streptomyces griseus* in 1944, immense and various antimicrobial and antitumor products produced by *Streptomyces* have gained worldwide scientific consideration [7]. Generally, 80% of discovered antibiotics are produced by the genus *Streptomyces* and rare *Actinobacteria*, such as *Actinomadura*, and only 20% are synthesized by fungal species [8]. Actually, genomes of *Streptomyces* species can possess a panel of biosynthetic gene clusters (BGCs) responsible for the production of various secondary metabolites with antimicrobial activities [9–11].

Resistance to antibiotics remains a serious global health issue, and there is an urgent need for novel and effective antibiotics. By the end of the last century, despite the extensive efforts in screening soil-dwelling* Actinobacteria* for new bioactive compounds for pharmaceutical use, most attempts went in vain [12]. As a result, investigating *Actinobacteria* living in unexplored habitats such as desert soils, marine biotopes, saline environments, and other extreme environments, in which certain physical and/or chemical factors differ significantly from those in natural and well-studied habitats, has shown great importance because of the novelty of their antimicrobial compounds [13, 14].

Morocco, with its distinctive climate and geographical position, represents a multitude of diverse ecological niches, many of which are considered extremes, such as saline biotopes, arid deserts, thermophilic sources, and polymetallic mines [15–17]. However, scarce data are available on the diversity and ecological roles of microorganisms and, more precisely, *Actinobacteria* living in the such ecosystems [18–23].

Lead mines (Ahouli-Zaida-Mibladen), located in Midelt, Morocco, were considered as the biggest Moroccan Pb mining sites of the last century. Currently abandoned, they remain largely unexplored and unexploited. To the best of our knowledge, no prior study has reported the diversity and antimicrobial effects of *Actinobacteria* living in these mining ecosystems. Thus, the present work aims firstly to isolate and characterize *Actinobacteria* from extreme mines and secondly to analyze the distribution and the antimicrobial potential of those isolates.

## 2. Materials and Methods

### 2.1. Description of the Studied Sites, Sampling, and Chemical Analyses

**Figure 1:**
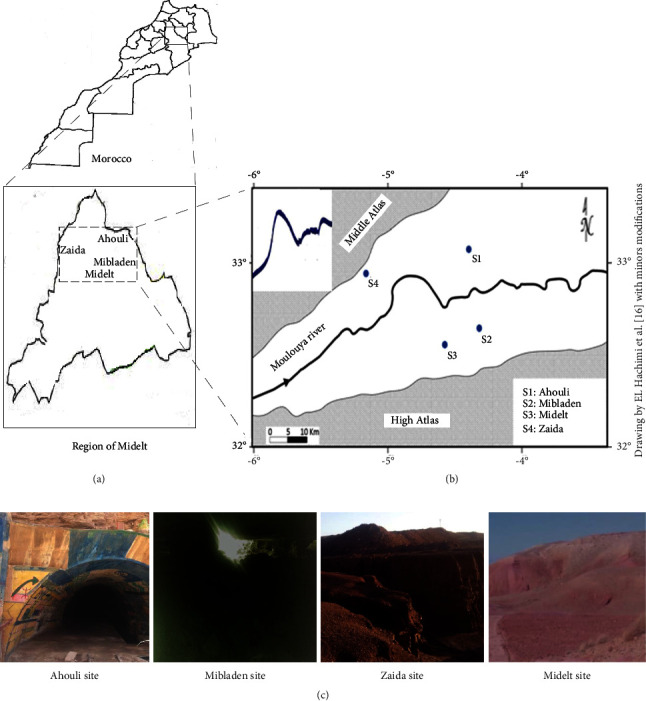
(a) Map of sampling sites of *Actinobacteria* isolation from the region of Midelt, between the Middle and High Atlas mountain ranges, in Morocco. (b) Positioning of the sampling sites of this study on the map drawn by El Hachimi et al. [16]. (c) Pictures of the sampling sites related to the present study.

Soil samples (*n* = 16) were collected from four different abandoned mining sites located in High Moulouya, in the province of Midelt, Morocco (Figure 1). The studied sites are located at Ahouli (coordinates; 32°48′45″N, 04°35′11″W, alt.1130 m), Mibladen (coordinates; 32°45′08″N, 04°39′23″W, alt. 1130 m), Zaida (coordinates; 32°49′28″N, 04°57′18″W, alt. 1490 m), and Midelt (coordinates; 32°41′06″N, 4°44′42″W, alt. 1508) (Figure 1).

Four distinct locations within an area of 20 m^2^ were determined for sampling in each site. Each soil sample was collected at 5 to 10 cm depth in sterilized plastic bags and stored at 4°C for further analyses. For chemical analyses, samples were mixed to generate a composite soil and sieved until a fine powder was obtained.

One gram of each soil sample was used to determine organic matter content. Soil samples were dried in an oven at 500°C for 4 h. After drying, each sample was weighed to determine the lost weight [24]. Soil pH was measured with a calibrated pH 210 Microprocessor pH meter (Hanna Instruments, Romania). Five grams of soil sample were mixed with 50 mL of distilled water. After shaking for 20 min, the suspension was left to settle for 2 hours. The concentrations of 10 metals, Cd^2^^+^, Co^2^^+^, Cr^2^^+^, Cu^2^^+^, Fe^2^^+^, Mg^2^^+^, Na^+^, Ni^2^^+^, Pb^2^^+^, and Zn^2^^+^, were determined by inductively coupled plasma atomic emission spectroscopy (ICP-AES) using a spectrometer (Activa Horiba Jobin Yvon-Ovou 1048, France) after mineralization according to the method recommended by Alsac [25]. An amount of 0.5 g of the soil powder was mixed with 6 mL of hydrochloric acid and 2 mL of nitric acid. The mixture obtained was incubated at 95°C for 75 min, and the volume of the obtained filtrate was adjusted to 50 mL prior to ICP-AES analysis. The concentration values were expressed in mg/kg (metal/sol).

### 2.2. Culture Media

Starch casein agar (SCA) medium (soluble starch 10.0 g, casein 0.3 g, KNO_3_ 2.0 g, NaCl 2.0 g, K_2_HPO_4_ 2.0 g, MgSO_4_.7H_2_O 0.05 g, CaCO_3_ 0.02 g, FeSO_4_.7H_2_O 0.01 g, agar 20.0 g, and distilled water 1000 mL; pH 7.0 ± 0.2) was used for the isolation and the screening of antimicrobial activities among *Actinobacteria*. Bennett's medium (glucose 10.0 g, casamino acids 2.0 g, yeast extract 1.0 g, meat extract 1.0 g, agar 18.0 g, and distilled water 1000 mL; pH 7.0 ± 0.2) and Glucose/Yeast extract/Malt extract (GYM) medium (glucose 10.0 g, yeast extract 4.0 g, malt extract 10.0 g, and distilled water 1000 mL; pH 7.0 ± 0.2) were used for the screening of antimicrobial activities of *Actinobacteria*. International *Streptomyces* Project (ISP2) medium (malt extract 10.0 g, yeast extract 4.0 g, glucose 4.0 g, agar 18.0 g, and distilled water 1000 mL; pH 7.0 ± 0.2), ISP1 medium (yeast extract 3.0 g, peptone 5.0 g, agar 18.0 g, and distilled water 1000 mL; pH 7.0 ± 0.2), and ISP7 medium (glycerol 15.0 g, L-tyrosine 0.5 g, L-asparagine 1.0 g, K_2_HPO_4_ 0.5 g, MgSO_4_.7H_2_O 0.5 g, NaCl 0.5 g, FeSO_4_.7H_2_O 0.01 g, agar 18.0 g, distilled water 1000 mL, and trace salt solution 1 mL, pH 7.0 ± 0.2) were used for carrying out the morphological studies. ISP9 medium (Pridham and Gottlieb trace salts) (CuSO_4_. 5H_2_O 0.64 g, FeSO_4_.7H_2_O 0.11 g, MnCl_2_.4H_2_O 0.79 g, ZnSO_4_.7H_2_O 0.15 g, agar 18.0 g, and distilled water 100 mL) was used to assess the carbon source utilization.

### 2.3. Isolation of *Actinobacteria*

The isolation of *Actinobacteria* was carried out using the spread plate method by serially diluting 1 g of each sample in 9 mL of distilled water using SCA as a culture medium. Prior to isolation, suspended soil samples were incubated in a shaking incubator at 30°C for 30 min and 250 rpm, and then the suspension was sonicated 3 times for 30 s ON and 30 s OFF at 60 kHz. Dilutions up to 10^−6^ were prepared from each soil sample and 100 *μ*L of each dilution was plated onto the SCA medium. The agar plates were incubated at 30°C and examined every 2 days for microbial growth for up to 15 days. Morphologically distinct colonies with a special earthy odor were purified through repeated subculturing, and then sporulating and non-sporulating pure isolates were stored in sterile glycerol (20%, v/v) at −80°C.

### 2.4. Morphological, Chemotaxonomical, and Biochemical Studies

#### 2.4.1. Morphological Study

Morphological and cultural characteristics, such as the pigmentation of aerial and reverse substrate mycelia, and the presence of the diffusible pigments and melanoid pigments were examined after one week of growth using ISP2, Bennett's, ISP1, and ISP7 media [26]. Micromorphology and sporulation of isolates were examined under the light microscope (Olympus, Japan).

#### 2.4.2. Chemotaxonomical Study

For the chemotaxonomical study, the isolates were cultured in shake flasks (150 rpm, 30°C, 7 days) using modified Bennett's medium broth (glucose 10.0 g, yeast extract 1.0 g, meat extract 1.0 g, and distilled water 1000 mL; pH 7.0 ± 0.2). The dried biomass of each isolate was obtained by centrifugation at 9000 g. It was washed 3 to 5 times until complete elimination of any trace of culture medium before being well dried overnight at 45°C.

Regarding the diagnostic isomers of diaminopimelic acid (DAP), the dried biomass (10 mg) was hydrolyzed for 18 h with 1 mL of 6 N HCI in a sealed tube by incubation at 100°C in a sand bath. After cooling, the mixture was filtered through paper filter. The liquid hydrolysate was dried at 40°C, washed with distilled water to remove most of the HCl, and then dried again at 40°C. The remaining residue was solubilized in 0.3 mL of distilled water, and 20 *µ*L of the mixture was spotted on Whatman no. 1 paper with the amino acid standards [27]. Thin-layer chromatography (TLC) was performed for 4 hours by elution with methanol-water-10 N HCl-pyridine (80 : 17.5 : 2.5 : 10). Amino acids were detected by spraying the paper with acetone ninhydrin solution (0.1%, w/v), followed by heating for 2 min at 100°C. Diaminopimelic acid spots were green-olive fading to yellow, and thus the LL form of DAP acid migrates faster than the DL form. Other amino acids, such as glycine, in the mixture gave purple-pink spots and moved faster than the diaminopimelic acid [27].

As for the whole-cell sugar pattern, the dried biomass (25 mg) was hydrolyzed for 2 h with 1.5 mL of 1 N sulfuric acid in a sealed tube and incubated in a boiling water bath. After cooling, the obtained hydrolysate was poured into a conical centrifuge tube, and saturated barium hydroxide solution was added by drops until the pH reached about 5.2–5.5. The supernatant, obtained after centrifugation at 9000 g, was evaporated to dryness, and the residue was redissolved in 0.3 mL of distilled water. A volume of 1 *µ*L of the mixture was deposited on the base line of a cellulose plate (DC-Fertigfolien, F 1440, Cellulose) with 1 *µ*L of two solutions containing standard sugars. The first solution contains L-rhamnose, L-mannose, D-glucose, and D-ribose at 1%, and the second one consists of D-galactose, L-arabinose, and D-xylose, each at 1% as well [28]. TLC was performed with an elution system composed of n-butanol-distilled water-pyridine-toluene (10 : 6:6 : 1) for approximately 4 h. The spots were developed by spraying the plate with acid aniline phthalate solution (3.35 g phthalic acid solubilized in 100 mL of water-saturated butanol plus 2 mL of aniline) and dried at 100°C for 5 min. The hexoses (glucose, galactose, and mannose) appeared with a yellow color and the pentoses (ribose, xylose, and arabinose) developed a pink color after drying [28].

#### 2.4.3. Biochemical Study

Biochemical and physiological characteristics were evaluated according to Williams et al. methods [29]. The assimilation of carbohydrate carbon sources (D-glucose, D-fructose, D-galactose, L-arabinose, myo-inositol, D-mannitol, D-raffinose, D-lactose, D-maltose, and D-saccharose) was carried out in ISP9 agar medium supplemented with 1% of the studied carbohydrate.

The degradation of casein (1%, w/v, skimmed milk), tyrosine (0.5%, w/v), and starch (1%, w/v) was carried out in agar nutrient medium. Nitrate reduction was studied by the addition of 0.2 mL of Griess reagent to the culture carried out on a nitrate medium composed of nutrient broth supplemented with 0.1% (w/v) of KNO_3_. The appearance of red color indicates the reduction of nitrates. For the decomposition of urea, 10 mL of 15% of urea solution sterilized by filtration was added to 75 mL of sterile urea broth (KH_2_PO_4_ 10.0 g, Na_2_HPO_4_ 9.5 g, yeast extract 1.0 g, 0.04% of phenol red solution 20 mL, and distilled water 1000 mL, pH 6.7). The mixture was transferred aseptically into 2.5 mL volume sterile tubes and inoculated with the tested isolate. An alkaline reaction demonstrated the presence of urease. Catalase production was carried out by adding a few drops of H_2_O_2_ onto old colonies cultivated on Bennett's agar medium.

Analysis of the ability of isolates to grow on Bennett's agar medium at different temperatures (15, 37, and 42°C) was performed. All results of biochemical and physiological studies were recorded after incubation for 7 days at 30°C.

### 2.5. Molecular Identification

For DNA extraction, a bacterial pellet was prepared from a pure colony culture. The pellet was resuspended in 500 *µ*L of TE buffer (10 mmol/L Tris-HCl and 1 mmol/L EDTA; pH 8), and then 25 *µ*L of SDS (20%) was added. The mixture was incubated under stirring at 50°C for 10 min. After the incubation period, the mixture was centrifuged at 12000 g for 10 min. A 0.8 × V volume of isopropanol was added to the supernatant, and the mixture was centrifuged at 15000 g for 10 min at room temperature. The obtained pellet was washed twice with 75% ethanol, dried, and then resuspended in 50 *µ*L of distilled water.

Amplification of the 16S rDNA was carried out in a Mastercycler personal thermocycler (Germany) using the universal primers: 27F (AGAGTTTGATCCTGGCTCAG) and 1492r (GGTTACCTTGTTACGACTT). The reaction mix was prepared at a final volume of 20 *µ*L containing 2 *µ*L of Taq buffer (5*x*), 1.2 *μ*L of MgCl_2_ (25 mmol/L), 3 *μ*L of dNTPs (1 mmol/L), 0.1 *μ*L of each primer (100 *μ*mol/L), 0.2 *μ*L of Taq polymerase (5 U/*μ*L), 11.4 *μ*L of pure H_2_O, and 2 *μ*L of the extracted DNA.

Sanger sequencing was carried out using an ABI PRISM 3130XL Genetic Analyzer (Applied Biosystems) at the Innovation Center (USMBA, Fez, Morocco). The 16S rRNA gene sequences were compared with the GenBank database using BLASTN. Multiple sequence alignments were performed using ClustalW [30]. The neighbor-joining method was applied for the construction of the phylogenetic tree, and the analysis was conducted in MEGA11 [31, 32].

### 2.6. Antimicrobial Activities of *Actinobacteria* Isolates

Antimicrobial activities of *Actinobacteria* were determined using a qualitative double microbial layer method on three different culture media (Bennett's, CSA, and GYM). The indicator pathogens used for antimicrobial screening were *Bacillus subtilis* ATCC 6633, *Staphylococcus aureus* ATCC 29213, *Listeria innocua* CECT 4030, *Escherichia coli* K12, *Pseudomonas aeruginosa* ATCC 27853, *Dickeya solani* IP2222, *Pectobacterium brasiliensis* 13471a, and fluconazole-resistant *Candida albicans* strain ATCC 10231.

Each isolate was spot deposited on culture media, and the agar plates were incubated at 30°C for 7 days. Afterward, these plates were covered with a 5 mL soft agar (0.5% agar) of either LB (Luria–Bertani) medium for bacteria or YPG (yeast extract-peptone-glucose) medium for *Candida* growth, preinoculated with 100 *µ*L from an overnight culture of the test microorganism (OD600 nm ≈ 0.1). These cultures were poured carefully on the surface of the plates and incubated during 24 h at 30°C for *C*. *albicans* and 37°C for the tested bacteria. Antimicrobial activity was assessed by measuring the diameter of the clear zone of growth inhibition (DI = mm). Each experiment was conducted in three independent replicates, and the mean value of the inhibition zone diameter was calculated.

### 2.7. Statistical Analysis

Data were expressed as mean ± standard deviation obtained from triplicate experiments. Significance of differences was analyzed using Tukey's test, a correlation matrix was performed under RStudio program version 4.0.2, clustering dendrograms were performed using RStudio program version 4.1.3, and the heatmap was performed under GraphPad Prism version 9.3.1 software.

## 3. Results

### 3.1. Soil Sample Characterization and Isolation of *Actinobacteria*

pH, organic matter, and trace metal elements were measured for each soil sample collected from the mining sites. The soil's pH average values varied from 7.08 (Zaida site) to 8.79 (Ahouli site), characterizing the soil as slightly alkaline (Table 1). Trace metal element concentrations varied considerably between samples from different sites but also between samples within the same site. This was mainly observed for Cr, Cu, Fe, Na, Pb, and Zn.

For all soil samples, except S1, S2, and S4 taken from the Midelt site, the content of Pb was greater than the normal world averages (35 mg/kg) for uncontaminated soils given by Bown [33] (*P* < 0.05). This content ranged from 47.97 to 113.86 mg/kg, 495.28 to 55623 mg/kg, and 131.80 to 634.69 mg/kg in Ahouli, Mibladen, and Zaida sites, respectively, compared to 35 mg/kg measured in the normal world averages (Table 1). Cd content was also higher than the normal world average (0.35 mg/kg) (*P* < 0.01) with average values ranging from 3.58 mg/kg in the Ahouli site to 5.15 mg/kg in the Zaida site. The average values of Pb and Cd exceeded the normal world averages (these values are 2.08, 11.10, and 69.01 folds increase in Ahouli, Zaida, and Mibladen sites, respectively, for Pb, and 10 to 14.73 folds increase for Cd in the four sites), revealing that the study areas, mainly Mibladen and Zaida sites, are highly rich in Pb and Cd metals (Table 1).

The assessment of organic matter revealed that the Zaida site exhibits a low content with an average of 1.66%. The maximum content of organic matter reached an average of 4.3% in the Ahouli site, followed by an average of 3.05 and 3.56% in the Mibladen and Midelt sites, respectively. The bacterial concentration determined in all sites varied considerably between 9.2 × 10^4^ and 2.28 × 10^8^ CFU/g of soil. One hundred and forty-five isolates were distributed over the isolation sites as follows: 41 in the Ahouli site, 19 in the Mibladen site, 25 in the Midelt site, and 60 isolates in the Zaida site (Table 1).

**Figure 2:**
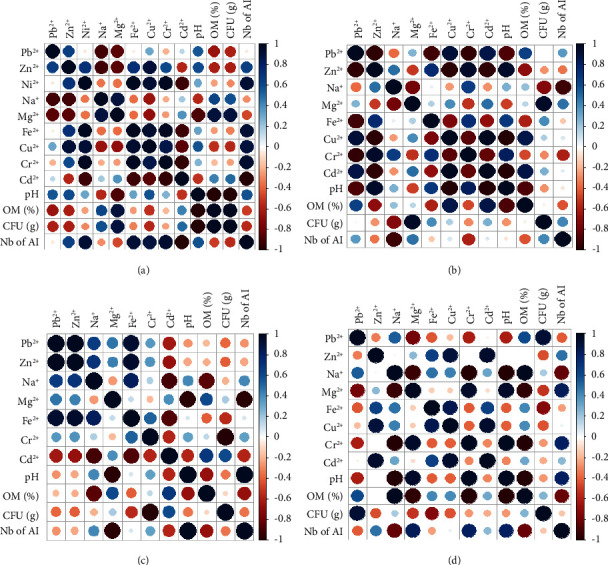
Correlation analysis between heavy metals, pH and organic matter of soil samples, and the bacterial concentration and *Actinobacteria* isolates in sites. (a) Ahouli. (b) Mibladen. (c) Midelt. (d) Zaida. OM: organic matter; CFU: colony-forming unit; Nb of AI: number of *Actinobacteria* isolates. Concentrations of Pb, Zn, Ni, Na, Mg, Fe, Cu, Cr, and Cd were given in mg/kg of soil.

A positive correlation was observed between the bacterial load and both Mg and Cd metals at the four studied sites. A negative correlation was observed between Pb and Zn metals and both bacterial load and number of *Actinobacteria* isolates in Ahouli and Midelt sites. However, in the Zaida site, a positive correlation was observed between Pb and the bacterial load. No influence of the Pb concentration was detected on the bacterial load and the *Actinobacteria* isolates in Mibladen site (Figure 2).

The influence of the other metal ions on the two parameters (bacterial load and *Actinobacteria* isolates) was variable in all sites. For organic matter, the influence of this parameter on both bacterial load and *Actinobacteria* isolates was variable in all samples with a preference for a high percentage of organic matter (Figure 2).

### 3.2. Phenotypic Analysis of *Actinobacteria* Isolates

The phenotypic characterization of the 145 actinobacterial isolates was carried out through the observation of *Actinobacteria*'s distinctive characteristics, such as colony pigmentation, diffusible and melanoid pigments, the color of aerial and substrate mycelia, spore chains, and aerial hyphae arrangement. Chemotaxonomical and classical biochemical tests were performed to better characterize the isolates.

**Figure 3:**
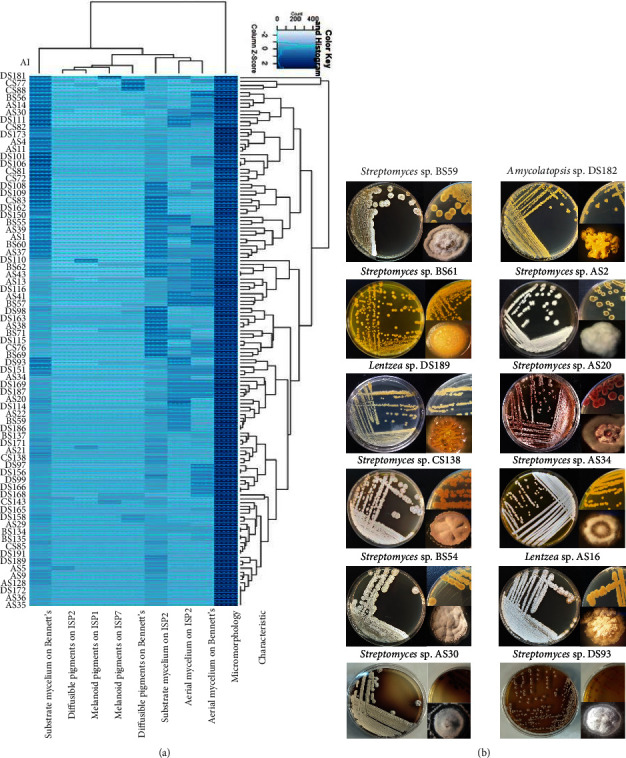
Diversity of culturable *Actinobacteria* isolated from lead mine sites. (a) Dendrogram showing the clustering of 145 actinobacterial isolates based on their morphological characteristics. AI: *Actinobacteria* isolate. (b) Phenotypes of some actinobacterial isolates: for each example, the front and back of the agar plate-grown bacteria are both denoted with the phenotype of a single colony.

**Figure 4:**
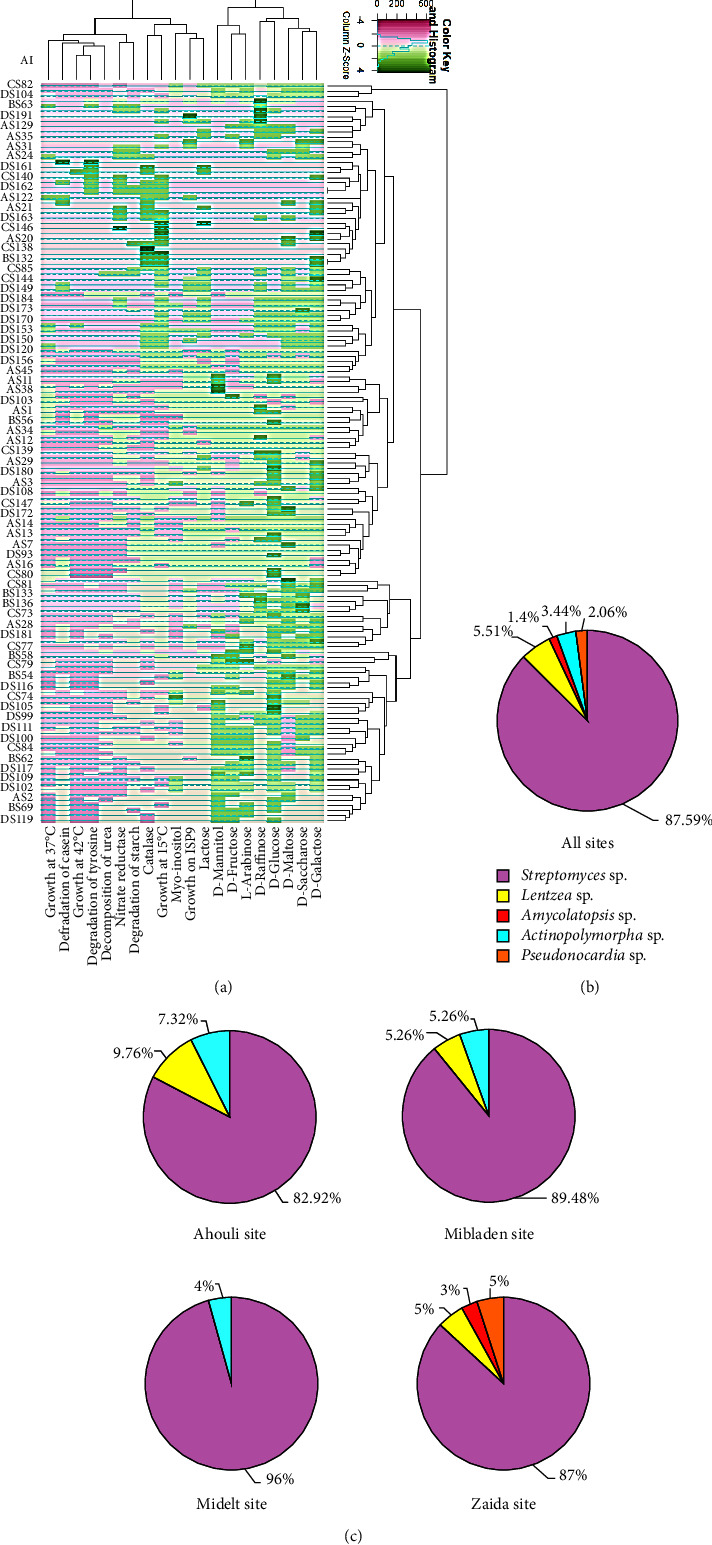
Diversity of culturable *Actinobacteria* isolated from lead mine sites. (a) Dendrogram showing the clustering of 145 actinobacterial isolates based on their biochemical characteristics. AI: *Actinobacteria* isolate. (b) Pie chart representation of the percentage frequency of actinobacterial genera in all sites and (c) within the total number of isolates in each site.

Based on the above mentioned tests, the 145 isolates were grouped into several clusters, each containing many subclusters, suggesting a potential diversity within the isolated *Actinobacteria*. Figures 3(a) and 4(a) illustrate the clustering according to the morphological and biochemical studies, classifying the isolates into more than 12 groups.

According to Bergey's Manual of Systematic Bacteriology [34], phenotypic characteristics positioned the isolates at the genus level. Most isolates grew well on Bennett's and ISP2 agar plates and showed well-developed aerial hyphae and a good sporulation on both media. They also produced a wide range of pigments (responsible for the color of substrate and aerial mycelia) and can produce diffusible pigments ranging from yellow to brown-red (Figure 3(b)). The aerial mycelium, which rarely fragments, forms short, medium, or long spore chains. Some isolates were able to hydrolyze starch, produce nitrate reductase, and show good growth on specific carbohydrates as the only source of carbon.

The chemotaxonomical study of isolates showed that their peptidoglycan cell contained LL-isomer of DAP in addition to the glycine, glutamic acid, and alanine for most isolates. Characteristic sugars in the whole-cell hydrolysates were rarely present and even absent for most isolates, and these properties are characteristic of *Streptomyces*, which represent 87.59% of all isolated *Actinobacteria*. The other isolates (no *Streptomyces*) contained in their peptidoglycan the DL isomer of DAP with or without glycine. In addition, they contained some characteristic sugars such as the combination of the three sugars: galactose, arabinose, and mannose or the combination of the two sugars: galactose and arabinose.

All the gathered data showed that the 145 actinobacterial isolates belong to five bacterial genera, which are *Streptomyces*, *Amycolatopsis*, *Pseudonocardia*, *Lentzea*, and *Actinopolymorpha*. All the studied sites contain *Streptomyces* as an abundant genus (Figure 4(b)).

The analysis of the results illustrated in Figure 4(b) showed that the Ahouli and Mibladen sites contain, in addition to *Streptomyces*, other bacterial genera: *Lentzea* and *Actinopolymorpha*, with a percentage of 9.76% and 7.32% in Ahouli site and 5.26% for each genus in Mibladen site, respectively. Midelt site contains only one isolate affiliated to the genus *Actinopolymorpha* (4%), and Zaida site contains 3 genera: *Amycolatopsis* (3%), *Pseudonocardia* (5%), and *Lentzea* (5%) (Figure 4(c)). It should be noted that genera *Amycolatopsis* and *Pseudonocardia* were present only in Zaida site, characterized by its high content mainly in sodium (Na), which indicates the halophilic trait of these genera (Figure 4(c)).

### 3.3. Phylogenetic Analysis

Twenty-five strains with important antagonistic activities and five strains (AS20, BS56, CS86, DS182, and DS189) without antimicrobial activity were identified up to the species level using the 16S rRNA gene fragment and considered for carrying out the phylogenetic analysis. To determine the relatedness among the selected strains, 16S rRNA gene sequences were aligned with the related sequences from the GenBank database.

The results showed that strains belong to two families *Streptomycetaceae* and *Pseudonocardiaceae*. With regard to the sampling site, the majority of strains were identified as *Streptomyces*: 35.29% from Zaida site, 29.41% from Ahouli site, 26.47% from Mibladen site, and 8.82% from Midelt site. Strains affiliated to *Amycolatopsis* (2.94%) were only obtained from the Zaida site. Nevertheless, strains of the genus *Lentzea* (5.88%) were found in Ahouli and Zaida sites (Table 2).

**Figure 5:**
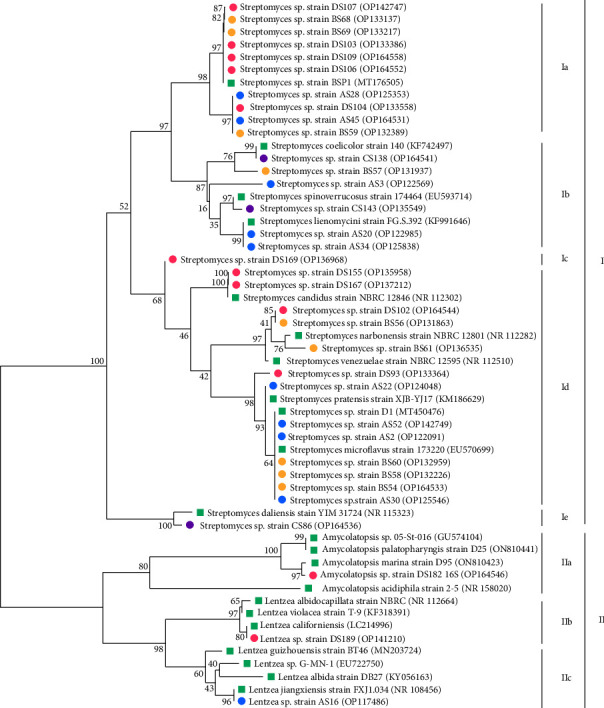
Neighbor-joining phylogenetic tree based on 16S rRNA gene sequences of selected strains (antagonistic strains with high antimicrobial activity and other inactive strains). Blue, orange, purple, and pink circles represent strains from, respectively, Ahouli, Mibladen, Midelt, and Zaida sites. Red square represents GenBank closest type species. Number at branches designates bootstrap values of neighbor-joining method from 1000 replicates and the bar designates 1% sequence divergence. I and II: clades I and II; Ia, Ib, Ic, Id, and Ie: subclades Ia, Ib, Ic, Id and Ie; IIa, IIb, and IIc: subclades IIa, IIb, and IIc. The evolutionary distances were calculated using the maximum composite likelihood method, and the analysis was conducted in MEGA11 [31, 32].

The neighbor-joining method was applied for the construction of the phylogenetic tree. Based on the results obtained, the sequences of *Actinobacteria* strains were divided into two major clades (clade I and clade II) (Figure 5). Clade I was formed by the majority of strains belonging to the genus *Streptomyces* and diverged into five subclades with a certain geographical link. Subclades Ia and Id were the largest and were clustered from sequences of strains obtained from Ahouli, Mibladen, and Zaida sites (Figure 5). Subclade Ia is composed of sequences from strains of Mibladen and Zaida sites, very close to each other with an identical bootstrap value, and only two sequences from Ahouli site are grouped with two other strains each belonging to the first two sites. Thirteen other strains from the Ahouli (four strains), Mibladen (five strains) and Zaida (four strains) sites formed the subclade Id within the genus *Streptomyces*. Approximately fifty percent of these strains, from Ahouli and Mibladen sites, have an identical bootstrap value (Figure 5). Subclade Ib presented sequences from strains of Ahouli and Midelt sites and only one sequence from Mibladen site. Furthermore, it clustered two strains with the same bootstrap value: AS20 without activity and AS34 with activity (Figure 5). Clades Ic and Ie were the smallest, with only one sequence for each clade: DS169 in clade Ic and CS86 in clade Ie (Figure 5). Strains in the genera *Lentzea* and *Amycolatopsis* were clustered to subclades IIa, IIb, and IIc to create clade II. The clade sequence IIa of *Amycolatopsis* DS182 was detected exclusively in Zaida site. Similarly and finally, the clades IIb and IIc only appeared in Ahouli and Zaida sites (Figure 5).

### 3.4. Antimicrobial Activities

**Figure 6:**
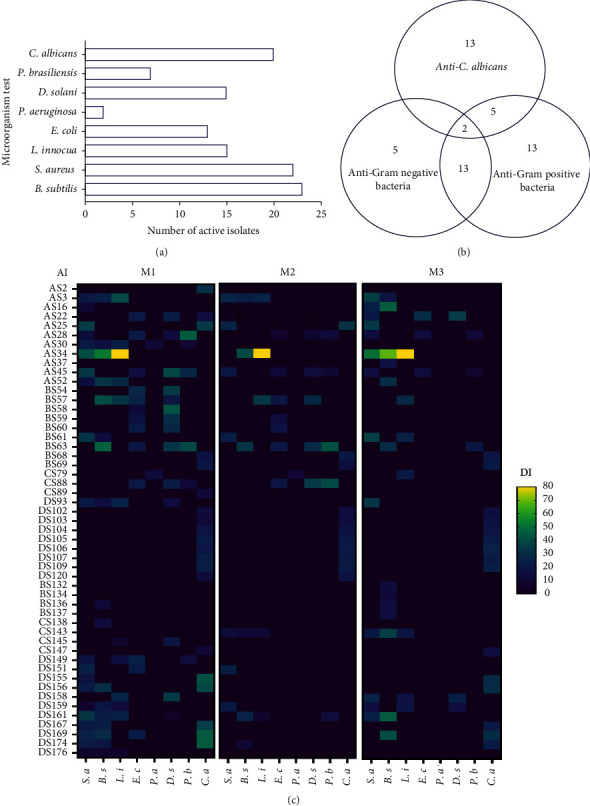
Antimicrobial activities. (a) Number of *Actinobacteria* isolates with antimicrobial activity broken down by indicator microorganisms (detected in a mutually exclusive way on the three culture media). (b) Venn diagram summarizing the spectrum of activity of the 51 antimicrobial producing actinobacterial isolates. (c) Heatmap summarizing antimicrobial activities. M1: on Bennett's medium; M2: on GYM medium; M3: on CSA medium; DI: diameter of the inhibition zone (given in mm); AI: *Actinobacteria* isolate; *S*. *a*: *S*. *aureus*; *B*. *s*: *B*. *subtilis*; *L*. *i*: *L*. *innocua*; *E*. *c*: *E*. *coli*; *P*. *a*: *P*. *aeruginosa*; *D*. *s*; *D*. *solani*; *P*. *b*: *P*. *brasiliensis*; *C*. *a*: *C*. *albicans*.

The potential of each isolate to produce molecules with antimicrobial activity against both Gram-positive and Gram-negative bacteria and yeast (*C*. *albicans*) was assessed via double layer assay using three different culture media (Bennett's, GYM, and CSA) (Figure 6). Results indicate that among the 145 tested isolates, 51 (35.17%) exhibited antimicrobial activities against at least one test microorganism. Out of these 51 isolates, 13 (25.49%) displayed a positive activity against *C*. *albicans*, 31 (60.78%) inhibited Gram+ and/or Gram^−^ bacteria, and 7 (13. 72%) isolates displayed a positive activity against both bacteria and *C*. *albicans*.

The strongest inhibitory activities were noted against Gram-positive bacteria (for 23 and 22 active isolates against *B. subtilis* and *S. aureus*, respectively) and the yeast *C. albicans* (for 20 active isolates) as compared to those tested against Gram-negative bacteria (Figure 6(a)).

As shown in the Venn diagram (Figure 6(b)), most of the strains produced a narrow spectrum of antimicrobial compounds. Of 51 active strains, 31 (60.78%) antimicrobial producing strains seemed to be active against a specific category of microorganisms: Gram-positive bacteria (13 strains), Gram-negative bacteria (5 strains), or *C. albicans* (13 strains) (Figure 6(b)). Eighteen strains (35.29%) could be characterized as producers of a middle spectrum of activity for both, Gram-positive and Gram-negative bacteria inhibition (13 strains) or Gram-positive bacteria and *C. albicans* inhibition (5 isolates) (Figure 6(b)). Only two (3.92%) strains, *Streptomyces* sp. AS22 and *Streptomyces* sp. DS169 could be characterized as producers of a large spectrum of activity against some of the tested Gram-positive and Gram-negative bacteria, as well as the yeast *C. albicans* (Figure 6(b)).

Regarding their antimicrobial potentials, ten strains (AS3, AS22, AS28, AS34, AS45, BS57, BS61, BS63, CS88, and DS158) displayed highly remarkable activities. These activities were expressed depending on the culture medium, the actinobacterial isolate, and the type of the tested strain.

Three *Streptomyces* strains (AS3, AS34, and BS61) were found to be remarkably active against all tested Gram-positive bacteria (*S. aureus*, *B. subtilis*, and *L. innocua*). Strain AS34 had a high inhibitory effect on the growth of three tested Gram-positive bacteria with inhibition zones ranging from 36.50 ± 09.26 to 80.25 ± 01.36 mm in all tested media (Figure 6(c)).

It is worth mentioning that BS61 lost its activity against *L. innocua* when cultivated on GYM and Bennett's media. In addition, strains AS28, AS45, BS57, and BS63 showed a high production of antimicrobial compounds against Gram-negative and Gram-positive bacteria in both Bennett's and GYM media as compared to the CSA medium (Figure 6(c)).

The strain BS57 displayed a medium-dependent inhibition specificity. It was active against *B. subtilis* solely in Bennett's medium, exhibited activity against *E. coli* and *D. solani* in Bennett's and GYM media and inhibited *L. innocua* in all tested media. The strain BS63 displayed a middle spectrum of activity against both Gram-positive and Gram-negative bacteria. It was more active against the phytopathogenic bacteria (*P. brasiliensis* and *D. solani*) in the GYM medium with 42.38 ± 00.65 and 29.83 ± 04.65 mm inhibition zones towards *P. brasiliensis* and *D. solani*, respectively. However, the strain was inactive against *E. coli* and *D. solani* when cultivated on the CSA medium (Figure 6(c)).

The strain DS169 inhibited the growth of *C. albicans* when grown on Bennett's and CSA media and was able to inhibit the growth of *S. aureus* only on Bennett's medium. Four isolates (BS68, BS69, DS106, and DS107) were similarly active only against *C. albicans* on the three used culture media (Figure 6(c)).

**Figure 7:**
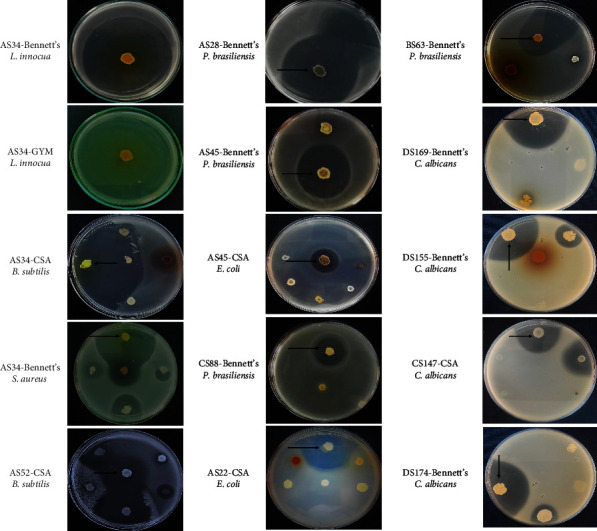
Selected representative examples of isolates showing antimicrobial activities. Isolates codes-media, and tested microorganisms are noted and the arrow indicates the active strain.

Representative agar plate pictures illustrating selected examples of the antagonistic activities are shown in Figure 7.

## 4. Discussion

The Ahouli-Mibladen-Zaida mines were the largest Moroccan Pb-Zn mining districts of the last century. They had been discovered in 1916 and were closed back in 1986. A hundred-year period could have been sufficient for the development and the adaptation of microorganisms to severe living conditions.

In line with prior research on the assessment of trace metal contamination levels in sediments and water from these mining sites [16, 35, 36], we have been exploring extreme environments (the Dead Sea in Jordan and hypersaline sites in the Pre-Rif region in Morocco) [22, 37] for the isolation of bacteria of biotechnological potential. We were the first group searching for *Actinobacteria* from Ahouli-Mibladen-Zaida mining areas and determining their potential to produce antimicrobial agents.

From the data analysis, we noted that the bacterial load and the number of *Actinobacteria* isolates inside samples decreased along with the increase of Pb concentration in the Ahouli and Midelt sites. In the Zaida site, the bacterial load is proportional to Pb concentration. We speculate that this increase or decrease might be due to the ability of bacteria to adapt (Mibladen and Zaida sites) or not (case of Ahouli and Midelt sites) to heavy metals in the different mining sites. In accordance with our findings, prior studies have shown that heavy metals in lead-zinc mines decrease microbial diversity by increasing *Proteobacteria* and by decreasing *Actinobacteria* and other phyla [38–41]. Other studies demonstrated that *Actinobacteria* and *Proteobacteria* were predominant in heavy metal contaminated soils [42–45]. Therefore, the present study on actinobacterial isolates from mining sites in the region of Midelt, Morocco, will be considered as an adding value to the previous researches on *Actinobacteria*.

Phenotypic analysis based on morphological, chemotaxonomical, and biochemical characteristics affiliated the *Actinobacteria* isolates to five genera, including *Streptomyces*, *Pseudonocardia*, *Lentzea*, *Amycolatopsis*, and *Actinopolymorpha*. We have also observed some differences at the genus level between sites: isolates of the genus *Actinopolymorpha* were found only in soil samples of Ahouli and Mibladen, while *Amycolatopsis* and *Pseudonocardia* were identified only within the Zaida mining site. Isolates affiliated to these latest genera or to the genus *Lentzea* were found in the soil samples of Ahouli, Mibladen, and Zaida sites, which present higher concentrations of Pb when compared to the Midelt site where *Streptomyces* was predominant, with only one isolate belonging to the genus *Actinopolymorpha*. This indicates that the chemical characteristics, mainly the high Pb concentration, might be favorable to these bacterial genera. However, the *Amycolatopsis* and *Pseudonocardia* genera were identified as habitat-specific genera since they were specifically found only in the Zaida site. These differences could also be attributed to the specific physicochemical and biological characteristics of each mining site.

The most abundant genus from all sites was *Streptomyces* (87.59%) followed by *Lentzea* (5.51%). This finding goes in line with previous studies showing that *Streptomyces* is the most abundant microorganism in heavy metal natural soils and other heavy metal contaminated and uncontaminated soils [46–49]. It has also been reported that several *Streptomyces* strains were resistant to heavy metal elements [47–53]. The rare genera belonging to the *Pseudonocardiaceae* family (*Amycolatopsis* and *Pseudonocardia*) found in our studied areas have been previously reported from other heavy metal soils and were reported to be resistant to certain metals [46, 47, 54, 55]. However, little is known about the *Lentzea* genus living in heavy metal ecosystems [56, 57], and no prior data have been reported on the isolation of *Actinopolymorpha* from heavy metal ecosystems.

Twenty-nine strains with high antimicrobial potential and other five inactive strains were characterized at the species level. All strains were classified within two families and three genera, indicating a partial diversity among mining *Actinobacteria*. The composition of *Actinobacteria* as obtained by the phylogenetic tree was more diverse as compared to *Actinobacteria* isolated from abandoned mining areas of Marrakech, Morocco [47]. *Streptomyces* strains fall under one major clade which is divided into five subclades (Ia–Ie), and rare *Actinobacteria Amycolatopsis* and *Lentzea* were assembled together to form a second major cluster. More than half of the strains displayed different phenotypic characteristics and bioactivity.

Regarding bioactivity, strains with closely similar 16S rDNA sequences showed different bioactivity profiles. For example, the strain AS20 (OP122985) from site Ahouli is without antimicrobial activity and is clustered with the strain AS34 (OP125838), from the same site, which is potentially active against Gram-positive bacteria. The same observation is noted for the inactive strain BS56 (OP131863) which is closer to DS102 strain (OP164544) which was active against *C. albicans* suggesting a potential diversity among strains from the same site.

In general, the phylogeny of *Streptomyces* strains showed a geographical-antimicrobial relationship between the subclades of clade I. Subclade Ia contained the majority of strains from Zaida and Mibladen sites displaying activity against only *C. albicans*, while subclade Id was composed of strains with different antimicrobial activities and isolated from three different sites. It is worth mentioning that the strain CS86 (OP164536), without antimicrobial activity, from the Midelt site, forms another subclade which differs from the major subclades. This suggests a potential diversity among *Streptomyces* strains. Rare *Actinobacteria* DS189 (OP141210) and AS16 (OP117486) of the genus *Lentzea* isolated from two different sites were clustered in two different subclades, and only AS16 displayed antimicrobial activity. Our results show that *Actinobacteria* strains display certain selectiveness towards tested pathogens, and that there is certainly a relationship between the antimicrobial capacities of these strains and their geographical site. This result is in line with the findings of previous studies [58].

Soil *Actinobacteria* have been largely studied, worldwide, for their production of antimicrobial agents. In Morocco, several studies have reported the isolation of *Actinobacteria* from many biotopes and demonstrated their antimicrobial potential. Recently, Rammali et al. highlighted the isolation of *Streptomyces* species from cold biotopes showing antimicrobial and antioxidant potentials [23]. Nafis et al. reported two new non-polyenic macrolide derivatives from *Streptomyces* Z26 isolated from a rhizospheric soil showing antifungal activity [59]. From the unexplored hot Merzouga desert, one hundred and sixty-three* Actinobacteria* isolates were obtained, of which 59% showed a high biological diversity with the greatest growth inhibition of pathogens tested [21]. The study reported by Oubaha et al. shows that *Streptomyces* species isolated from rhizospheric soils displayed a potent inhibitory effect against *Aphanomyces euteiches* causing the damping‐off disease of pea (*Pisum sativum* L.) [60]. However, *Actinobacteria* from Moroccan mining habitats have not been investigated for their biocontrol potential. This shows the interest of our study for the valorisation of *Actinobacteria* living in these biotopes by researching their antimicrobial potential.

From the obtained data, it was established that the majority of the 51 bioactive strains in all sites are potent antimicrobial strains and belonged to *Streptomyces* sp. For example, *Streptomyces* sp. AS34, from the Ahouli site, showed strong and specific antibacterial activity against Gram-positive bacteria. *Streptomyces* sp. AS22, from the same site, produces a large spectrum of activities with a mix of Gram-positive and Gram-negative bacteria and the yeast *C. albicans*. Similarly, previous reports have indeed shown that species of *Streptomyces* produce a variety of bioactive metabolites with both antibacterial and antifungal activities [23, 61, 62]. Moreover, other *Streptomyces* sp. strains (AS28, AS45, BS57, and BS58) and *Amycolatopsis* sp. DS158 exhibited relatively important activities against both Gram-positive and Gram-negative bacteria in line with previous studies that reported strains belonging to the *Amycolatopsis* genus exhibiting different antimicrobial compounds [63, 64].

Some strains affiliated to the *Lentzea* genus in the current study have antimicrobial activities that corroborate previous reports; this includes, for example, the recently reported strain *Lentzea tibetensis* sp. nov. that exhibits antimicrobial activity against Gram-positive bacteria and *Fusarium oxysporum* [65]. Antimicrobial activities in *Lentzea* indicate that rare *Actinobacteria* strains are appropriate sources of bioactive compounds. The recent analysis of the *Lentzea* genome reveals a repertoire of biosynthetic gene clusters (BGCs) among which more than 90% code for antimicrobial molecules including mainly terpenoids, lipopeptides, and thiopeptides [66].

## 5. Conclusion

The present study is the first of its kind to explore the diversity of actinobacterial isolates thriving in soils of mining areas with high Pb and Cd contents in the region of Midelt, Morocco. Data showed that the obtained *Actinobacteria* isolates were mainly assigned to five major genera (*Streptomyces*, *Amycolatopsis*, *Pseudonocardia*, *Lentzea*, and *Actinopolymorpha*). The genus *Actinopolymorpha* was, for the first time, isolated from a mining ecosystem. *Actinobacteria* isolates affiliated to *Streptomyces* genus exhibited a broad antimicrobial effect spectrum, indicating that mining biotopes are valuable sources of discovery for potential biocontrol *Actinobacteria* against human pathogen microorganisms.

## Figures and Tables

**Table 1:** Chemical characteristics, bacterial load, and number of *Actinobacteria* isolates of the soil samples collected from the studied sites.

	Ahouli site				Mibladen site				Midelt site				Zaida site			
	S1	S2	S3	S4	S1	S2	S3	S4	S1	S2	S3	S4	S1	S2	S3	S4
pH	8.26	7.45	8.79	8.05	8.69	7.65	8.20	8.72	7.94	7.28	7.78	7.78	7.76	7.08	7.90	7.95
Cd	3.79	3.89	2.72	3.95	4.74	4.94	4.44	4.30	4.29	4.45	4.18	4.46	4.44	6.73	4.76	4.70
Co	<1	<1	<1	<1	<1	1.75	<1	<1	<1	<1	<1	<1	<1	<1	<1	<1
Cr	13.96	13.82	21.41	11.56	13.32	8.20	19.98	16.77	11.30	10.93	11.27	10.55	8.72	8.67	9.23	9.10
Cu	9.51	6.42	22.29	11.31	44.95	251.34	30.75	14.03	<1	<1	<1	<1	<1	36.72	9.89	5.72
Fe	17696	16612	31231	15380	2847.3	873.74	6545.2	10378	8228.8	6379.4	10973	6173.9	8917.1	10754	8715.0	9633.4
Mg	5004.7	5009.4	5000.4	4999.6	4981.9	5004.7	4975.0	5008.4	4955.7	4984.1	4990.7	4982.4	5011.1	5014.5	5019.3	5020.9
Na	506.73	505.48	362.76	334.89	339.26	332.45	441.80	312.39	566.15	360.03	600.84	470.75	1803.6	1902.2	352.43	479.27
Ni	13.09	11.05	39.97	8.29	<1	<1	<1	<1	<1	<1	<1	<1	<1	<1	<1	<1
Pb	54.93	47.97	75.52	113.86	3024.7	5562.3	579.25	495.28	7.34	<1	91.21	1.08	634.69	359.80	428.98	131.80
Zn	25.25	24.66	38.06	33.36	16.84	12.77	18.96	17.13	16.73	15.56	24.00	15.37	13.70	25.88	21.47	17.19
OM (%)	2.33	10.84	2.28	1.76	2.48	4.56	3.30	1.89	1.97	5.61	3.59	3.10	2.13	2.4	1.21	0.93
CFU/g	1.6 10^6^	4.8 10^7^	1.47 10^6^	9.6 10^4^	6.78 10^6^	6.07 10^7^	3.59 10^5^	9.9 10^7^	2.57 10^7^	3.18 10^7^	2.48 10^7^	2.28 10^8^	2.12 10^8^	4.74 10^7^	1.77 10^8^	9.2 10^4^
Nb of AI	12	2	23	4	7	5	1	6	16	3	3	3	7	13	24	16

OM: organic matter; CFU: colony-forming unit; Nb of AI: number of *Actinobacteria* isolates; concentration of metals was expressed in mg/kg of soil; S1, S2, S3, and S4: samples 1, 2, 3, and 4.

**Table 2:** Identification of 29 antimicrobial potential *Actinobacteria* and 5 inactive strains^*∗*^ based on 16S rRNA gene sequences.

Strain No.	Site	NCBI GenBank accession number	GenBank closest known Species (accession number)	Similarity (%)	Identification
AS2	Ahouli	OP122091	*Streptomyces microflavus* (EU570699) *Streptomyces* sp. (KX950877)	99.93	*Streptomyces* sp.

AS3	Ahouli	OP122569	*Streptomyces africanus* (MT355846) *Streptomyces* sp. (MG930085)	99.72 99.44	*Streptomyces* sp.

AS16	Ahouli	OP117486	*Lentzea jiangxiensis* (NR_108456) *Lentzea sp*. (EU722750)	99.93 99.00	*Lentzea* sp.

AS20^*∗*^	Ahouli	OP122985	*Streptomyces lienomycini* (KF991646) *Streptomyces thinghirensis* (NR_116901)	99.93	*Streptomyces* sp.

AS22	Ahouli	OP124048	*Streptomyces* sp. (KT183568) *Streptomyces pratensis* (KM186629)	100	*Streptomyces* sp.

AS28	Ahouli	OP125353	*Streptomyces* sp. (MN826253) *Streptomyces albidoflavus* (MF663704)	99.79	*Streptomyces* sp.

AS30	Ahouli	OP125546	*Streptomyces* sp. (MT450476) *Streptomyces luridiscabiei* (MH241016)	100	*Streptomyces* sp.

AS34	Ahouli	OP125838	*Streptomyces lienomycini* (KF991646) *Streptomyces thinghirensis* (NR_116901)	100	*Streptomyces* sp.

AS45	Ahouli	OP164531	*Streptomyces* sp. (MT012002) *Streptomyces violascens* (MG190789)	100	*Streptomyces* sp.

AS52	Ahouli	OP142749	*Streptomyces* sp. (MT450476) *Streptomyces luridiscabiei* (MH241016)	100	*Streptomyces* sp.

BS54	Mibladen	OP164533	*Streptomyces mediolani* (OM980220) *Streptomyces anulatus* (AB184199)	99.85 99.64	*Streptomyces* sp.

BS56^*∗*^	Mibladen	OP131863	*Streptomyces venezuelae* (CP029197) *Streptomyces* sp. (KF772617)	99.71	*Streptomyces* sp.

BS57	Mibladen	OP131937	*Streptomyces caelestis* (NR_112512) *Streptomyces* sp. HQ385920	99.86 99.65	*Streptomyces* sp.

BS58	Mibladen	OP132226	*Streptomyces* sp. (MT450476) *Streptomyces flavofriseus* (MT355868)	100	*Streptomyces* sp.

BS59	Mibladen	OP132389	*Streptomyces* sp. (MK628999) *Streptomyces pratensis* (MH482882)	100	*Streptomyces* sp.

BS60	Mibladen	OP132959	*Streptomyces* sp. (MT450476) *Streptomyces pratensis* (KU870789)	100	*Streptomyces* sp.

BS61	Mibladen	OP136535	*Streptomyces* sp. (MN187461) *Streptomyces narbonensis* (NR_112282)	100 99.50	*Streptomyces* sp.

BS68	Mibladen	OP133137	*Streptomyces* sp. (MT176505) *Streptomyces hydrogenans* (MG195144)	100	*Streptomyces* sp.

BS69	Mibladen	OP133217	*Streptomyces* sp. (MT176505) *Streptomyces koyangensis* (MG188671)	100	*Streptomyces* sp.

CS86^*∗*^	Midelt	OP164536	*Streptomyces daliensis* (NR_115323) *Streptomyces cellulosae* (KP170478)	99.28 98.78	*Streptomyces* sp.

DS93	Zaida	OP133364	*Streptomyces* sp (KC414008) *Streptomyces cavourensis* (CP030930)	99.93 99.78	*Streptomyces* sp.

DS102	Zaida	OP164544	*Streptomyces* sp. (KF772617) *Streptomyces venezuelae* (NR_112510)	99.51 99.44	*Streptomyces* sp.

DS103	Zaida	OP133386	*Streptomyces* sp. (MT176505) *Streptomyces violascens* (MN704434)	100	*Streptomyces* sp.

DS104	Zaida	OP133558	*Streptomyces* sp. (MN826253) *Streptomyces albidoflavus* (MF663704)	100	*Streptomyces* sp.

DS105	Zaida	OP164568	*Streptomyces* sp. (MG893105) *Streptomyces lividans* (KU291358)	99.86	*Streptomyces* sp.

DS106	Zaida	OP164552	*Streptomyces* sp. (MT176505) *Streptomyces albidoflavus* (MH021968)	99.91	*Streptomyces* sp.

DS107	Zaida	OP142747	*Streptomyces* sp. (MT176505) *Streptomyces hydrogenans* (MG195144)	100	*Streptomyces* sp.
DS109	Zaida	OP164558	*Streptomyces* sp. (MT176505) *Streptomyces koyangensis* (MG188671)	100	*Streptomyces* sp.

CS138	Midelt	OP164541	*Streptomyces* sp. (KJ777675) *Streptomyces coelicolor* (KF742497)	99.43	*Streptomyces* sp.

CS143	Midelt	OP135549	*Streptomyces* sp. (MK608363) *Streptomyces spinoverrucosus* (EU593714)	100 99.71	*Streptomyces* sp.

DS155	Zaida	OP135958	*Streptomyces candidus* (MT760586) *Streptomyces candidus* (NR_112302)	99.92	*Streptomyces* sp.

DS167	Zaida	OP137212	*Streptomyces candidus* (NR_112302) *Streptomyces candidus* (NR_043504)	99.78 99.71	*Streptomyces* sp.

DS169	Zaida	OP136968	*Streptomyces* sp. (OL636374) *Streptomyces candidus* (JQ422145)	99.92	*Streptomyces* sp.

DS182^*∗*^	Zaida	OP164546	*Amycolatopsis marina* (ON810423) *Amycolatopsis palatopharyngis* (ON810441)	99.30	*Amycolatopsis* sp.

DS189^*∗*^	Zaida	OP141210	*Lentzea californiensis* (LC214996) *Lentzea violacea* (KF318391)	99.78	*Lentzea* sp.

^∗^Strains without antimicrobial activity.

## Data Availability

The data used to support the findings of this study are included within this article. Any required further information can be provided by the corresponding author upon request.
